# Fortifying Smart Home Security: A Robust and Efficient User-Authentication Scheme to Counter Node Capture Attacks

**DOI:** 10.3390/s23167268

**Published:** 2023-08-19

**Authors:** Iqra Asghar, Muhammad Ayaz Khan, Tahir Ahmad, Subhan Ullah, Khwaja Mansoor ul Hassan, Attaullah Buriro

**Affiliations:** 1 Department of Cybersecurity, Air University Islamabad, Islamabad 44000, Pakistan; iqraasghar064@gmail.com (I.A.); mansoor.hassan@mail.au.edu.pk (K.M.u.H.); 2 Department of Computer Science, Air University Islamabad, Islamabad 44000, Pakistan; ayaz.khan@mail.au.edu.pk; 3 Center for Cybersecurity, Brunno Kessler Foundation, 38123 Trento, Italy; 4 Faculty of Computer Science, National University of Computer and Emerging Sciences (NUCES-FAST), Islamabad 44000, Pakistan; subhan.ullah@nu.edu.pk; 5 Faculty of Engineering, Free University Bozen-Bolzano, 39100 Bolzano, Italy

**Keywords:** smart home security, user authentication, node capture attack

## Abstract

In smart home environments, the interaction between a remote user and devices commonly occurs through a gateway, necessitating the need for robust user authentication. Despite numerous state-of-the-art user-authentication schemes proposed over the years, these schemes still suffer from security vulnerabilities exploited by the attackers. One severe physical attack is the node capture attack, which allows adversaries to compromise the security of the entire scheme. This research paper advances the state of the art by conducting a security analysis of user-authentication approaches regarding their vulnerability to node capture attacks resulting in revelations of several security weaknesses. To this end, we propose a secure user-authentication scheme to counter node capture attacks in smart home environments. To validate the effectiveness of our proposed scheme, we employ the BAN logic and ProVerif tool for verification. Lastly, we conduct performance analysis to validate the lightweight nature of our user-authentication scheme, making it suitable for IoT-based smart home environments.

## 1. Introduction

The Internet of Things (IoT) has rapidly expanded, with many interconnected physical nodes exchanging data and information [1]. The growth of IoT devices is expected to reach approximately 38.6 billion connections by 2025 [1]. These devices find applications in both consumer and industrial domains, and the smart home environment is emerging as a prominent usecase [2,3,4]. Smart homes have numerous interconnected devices that enable remote users to manage and control their home appliances.

As the number of devices within IoT networks continues to increase, it becomes crucial to address management and security concerns for remote users. Security, in particular, poses a significant challenge for IoT networks, necessitating secure information exchange with attributes such as confidentiality, integrity, and availability to resist potential security attacks [5,6]. Among the security challenges in IoT, ensuring data privacy, authentication, authorization, and access control are critical [7,8]. Authentication, a fundamental security requirement, is especially challenging in smart home environments due to the resource-constrained nature of the devices [9,10].

To overcome these challenges and achieve secure user authentication, numerous user-authentication schemes for IoT-based smart home environments have been proposed in the literature. However, these authentication schemes often focus on general security attributes and neglect the threat of node capture attacks, particularly severe in resource-constrained devices within smart home environments. Without protection against node capture attacks, an adversary can compromise an authentication scheme, underscoring the need to design user-authentication schemes that provide mutual authentication and resist such attacks’ consequences.

**Figure 1:**
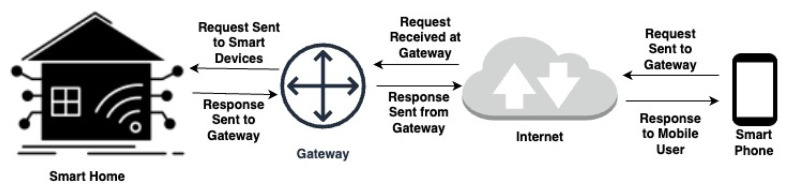
IoT-based smart home network.

The proposed scheme in this work is a lightweight authentication solution designed to address the security challenges faced in the smart home environment. Figure 1 illustrates a simplified IoT smart home environment architecture comprising smart devices, a gateway, and a mobile user. Smart devices can be tamper-proof or non-tamper-proof, collecting information for the mobile user in the environment. The gateway is a robust and tamper-proof node, bridging remote users (i.e., a participant who accesses and retrieves information from the deployed smart devices through the gateway) and smart devices. It is responsible for system monitoring and control. The proposed authentication scheme aims to provide user authentication, key agreement, sound repairability, no location tracking, user anonymity, forward secrecy, no password exposure, resistance to known attacks, and resistance to node capture attacks. The proposed scheme offers robust protection against unauthorized access and malicious activities by incorporating these essential security features. Furthermore, the scheme is designed to be computationally and communicationally efficient, making it suitable for resource-constrained environments.

The contributions of this paper are as follows, emphasizing the innovation brought forth by our study:

- We comprehensively analyze prevailing authentication mechanisms vulnerable to node capture attacks in IoT-based smart home environments. Our assessment identifies the shortcomings and security gaps present in these mechanisms.
- We introduce a novel user-authentication scheme designed to counter node capture attacks and fortify the security posture of IoT-based smart homes. This scheme is a pioneering response to the evolving threats in this domain.
- Our proposed scheme undergoes rigorous formal and informal analyses to validate its security strength. This ensures that our solution meets the stringent security requirements expected in smart home environments.
- We demonstrate a marked improvement in computation and communication costs compared to existing approaches through meticulous performance analysis. This efficiency enhancement is a significant advancement in IoT-based smart home security.

**Paper Organization**: The rest of the paper is organized as follows: Section 2 provides an overview of the existing literature on the topic. Section 3 explains the fundamental idea behind our proposed authentication scheme, emphasizing its lightweight nature. Section 4 discusses the underlying threat model for conducting formal and informal security analyses. Section 5 performs a security analysis of the suggested scheme to evaluate its robustness against potential attacks using BAN logic and ProVerif. Section 6 presents an informal security analysis of the proposed authentication scheme, further exploring its strengths and weaknesses. Section 7 reports on the performance evaluation of our approach, focusing on computation and communication costs. Section 8 summarizes our major findings and discusses potential future research directions.

## 2. Related Work

Many user-authentication schemes have surfaced in the literature in IoT-based smart home environments. These schemes share the goal of providing robust authentication mechanisms, although they exhibit varying effectiveness and security vulnerabilities. In this section, we explore the notable contributions in the field and present a meticulous comparative analysis of their respective schemes.

Vaidya et al. [11] proposed a password-based authentication protocol for smart homes, employing HMAC-based one-time passwords and smart card technology. They claim mutual authentication features and forward secrecy, which prevents the exploitation of stolen smart cards and clock-synchronization attacks. However, further analysis revealed vulnerabilities in their scheme, including susceptibility to password-guessing and user-impersonation attacks. Kim et al. [12] studied and analyzed these security vulnerabilities and proved that Vaidya et al.’s protocol is vulnerable to password-guessing and user-impersonation attacks. They proposed a solution incorporating hash-based one-time password algorithms and hash chaining to address these weaknesses. However, the Kim et al. scheme is still vulnerable to the same issues identified in Vaidya et al.’s protocol.

Li [13] proposed a key establishment scheme for secure smart home energy management systems. This scheme manages and stores multiple keys and certificates, enabling secure device communication. However, the scheme suffers from computation overhead due to the large number of keys and certificates it handles. Additionally, it lacks the crucial feature of mutual authentication between the user device and smart devices, leaving it susceptible to impersonation attacks. Similarly, Santoso et al. [14] designed an elliptic curve cryptography (ECC)-based user-authentication scheme for IoT-based smart home systems and addressed the issues of mutual authentication. Their protocol achieves mutual authentication between IoT devices and mobile users with the help of a central gateway node (GWN). However, their scheme does not provide user anonymity and untraceability features, making it vulnerable to insider attacks like smart card theft.

Similarly, Kumar et al. [15] also proposed a lightweight authentication and session key establishment protocol for IoT-based smart home systems. Their scheme claims resistance against notable attacks like key-stolen attacks. However, it does not provide mutual authentication between the mobile user and smart device and lacks user anonymity and untraceability features. The mutual authentication issues have been further addressed. Wazid et al. [16] designed a lightweight remote user-authentication protocol suitable for resource-constrained IoT-based smart home systems devices. The weakness in their scheme is reliance on a verification table on the GWN node for authentication purposes. This introduces other vulnerabilities that make the scheme susceptible to synchronization attacks. All the above schemes lack user anonymity and untraceability features.

Table 1 presents a comparative analysis of the discussed user-authentication schemes for IoT-based smart home systems. The table includes an evaluation of each scheme based on mutual authentication, user anonymity, untraceability, and identified vulnerabilities.

The comparative analysis shows that the existing user-authentication schemes for IoT-based smart home systems have various vulnerabilities and lack essential security features. Therefore, there is a need for an improved user authentication protocol that addresses these flaws and provides a higher level of security. In the following sections, we propose a novel and improved user-authentication scheme for IoT-based smart home systems, which mitigates identified vulnerabilities and enhances overall security. We also perform a detailed security analysis of our proposed scheme.

## 3. Threat Model

The threat model is an essential component of the security analysis, providing a clear understanding of the attacker’s assumptions and capabilities within the protocol’s context. In this section, we define the threat model based on the Dolev–Yao model [17] and outline the assumptions made regarding the adversary, referred to as Eve.

### Assumptions

- **Communication Interception**: Eve can intercept, inject, remove, or send new messages when two participants communicate over the public channel. This means that any information exchanged over the public channel is susceptible to manipulation or eavesdropping by Eve.
- **Parameter Understanding**: Eve can understand all the parameters exchanged over the public channel. This implies that Eve can analyze and comprehend the content of the messages transmitted between participants.
- **Attacker Identity**: Eve can be an outsider or a dishonest participant within the system. This encompasses the possibility of external attackers attempting to compromise the system’s security and internal attackers with insider knowledge or unauthorized access.
- **Gateway Security**: The gateway, which plays a crucial role in the protocol, is assumed to be a secure entity. This means Eve cannot compromise the gateway or gain unauthorized access to its resources or sensitive information.
- **Secret Parameter Protection**: Eve cannot access the secret parameters used in the protocol. These secret parameters are assumed to be securely transmitted between the relevant parties and are not accessible or known to Eve.

By outlining these assumptions, the threat model provides a clear understanding of the capabilities and limitations of the attacker within the proposed protocol. It helps identify potential vulnerabilities and design appropriate security measures to mitigate them.

## 4. Proposed User-Authentication Scheme

**Figure 2:**
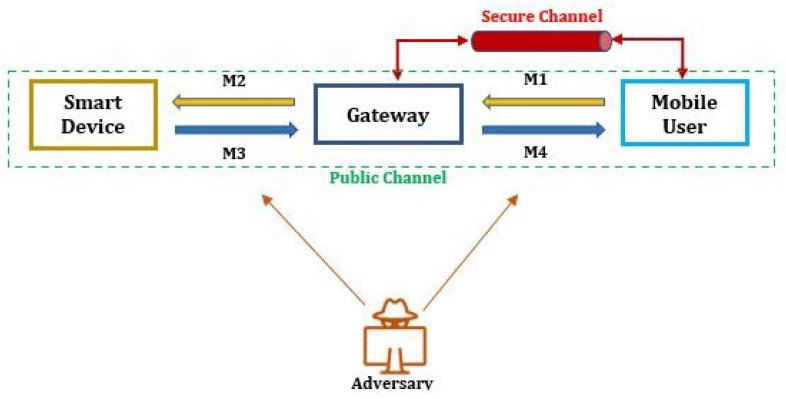
Proposed user authentication protocol.

The proposed protocol follows a general network model used in smart home environments, as depicted in Figure 1. Based on the analysis of state-of-the-art solutions, we have designed a user-authentication scheme to address the identified security vulnerabilities. Figure 2 presents the proposed user authentication protocol. Additionally, Table 2 provides a guide to the notations and abbreviations used in the protocol.

### 4.1. Assumptions

- During the pre-deployment phase of smart devices in the network, it is assumed that the gateway has shared its identity credential and the hash of the shared key $h(K_{GS})$ with the smart devices.
- Each smart device has a unique identity and a shared key $K_{GS}$ established between the device and the gateway.
- The identity of the gateway ($ID_{GW}$) is known to all participants.
- Every mobile user knows the identities of the smart devices.
- The gateway is considered a trusted entity within the smart home network.
- Both tamper-resistant and non-tamper-resistant smart devices are in the smart home network. Tamper-resistant devices are secure against node capture attacks, while non-tamper-resistant devices are vulnerable.
- The registration stage of the proposed protocol is carried out over a secure channel.
- The mobile user has the mechanism to extract and calculate location information and is capable of storing location history.

### 4.2. Stages of the Proposed Protocol

The proposed user authentication protocol consists of two stages:

#### 4.2.1. Registration Stage

**Figure 3:**
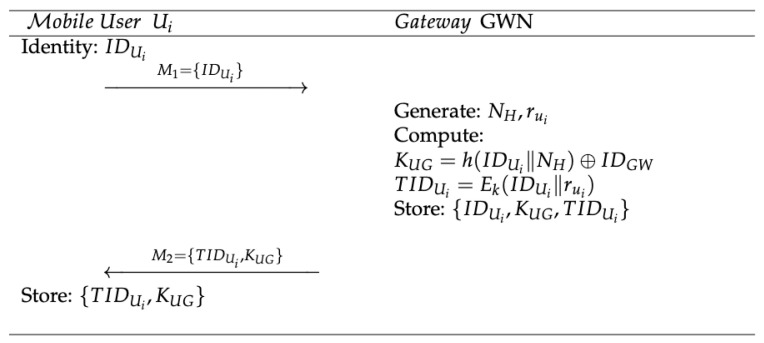
Registration stage of the protocol.

In the registration stage, the gateway issues security credentials to mobile devices. When a new mobile user ($U_{i}$) attempts to access a smart device, they must register the mobile device with the gateway. The registration process, illustrated in Figure 3, involves the following steps:

**Step 1:**  The new mobile user ($U_{i}$) submits their unique $ID_{U_{i}}$ to the gateway. $M_{1}=\left\{ID_{U_{i}}\right\}$
**Step 2:**  The gateway generates two random numbers ($N_{H}$ and $r_{u_{i}}$) and computes the shared secret key ($K_{UG}$) shared between the user and the gateway. The gateway also computes the temporary identity $TID_{U_{i}}$ by encrypting the user’s identity ($ID_{U_{i}}$) concatenated with the random number ($r_{u_{i}}$) using the secret key (*k*).
  $K_{UG}=h(ID_{U_{i}}\left|\right|N_{H})⊕ID_{GW}$
  $TID_{U_{i}}=E_{k}\left(ID_{U_{i}}∥r_{u_{i}}\right)$
**Step 3:**  The gateway stores and sends the message ($M_{2}$) to the requesting user ($U_{i}$).
  $M_{2}=\left\{TID_{U_{i}},K_{UG}\right\}$
  After receiving the message ($M_{2}$) from the gateway, the user stores it on their mobile device.

#### 4.2.2. Authentication Stage

**Figure 4:**
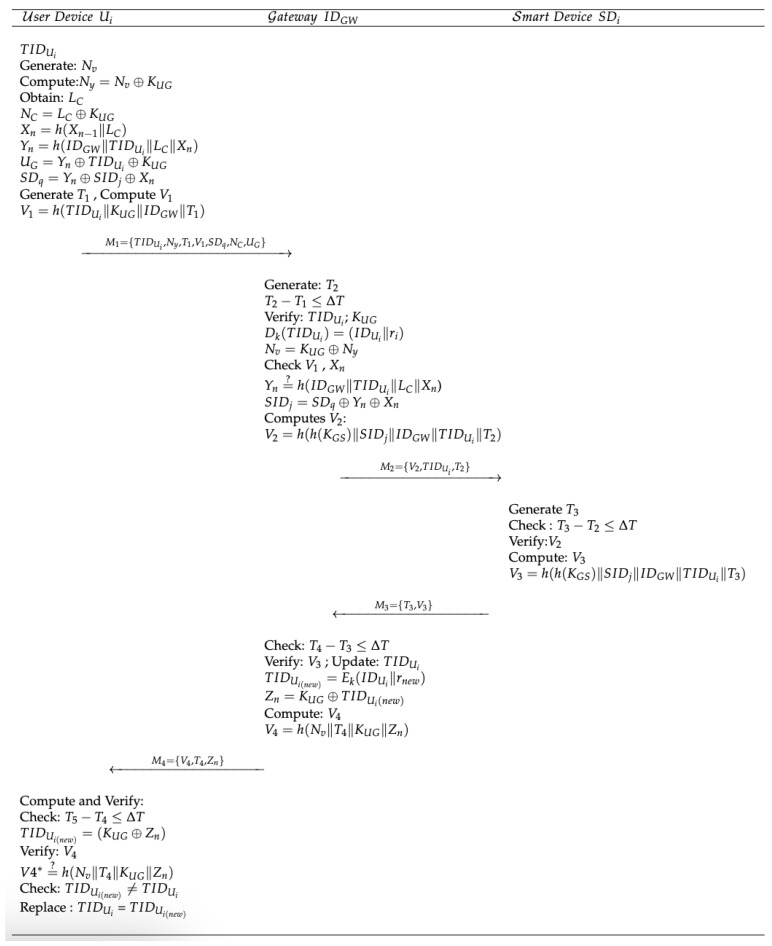
Proposed authentication scheme.

A registered mobile user can access a smart device after successful mutual authentication and establishing a session key with the smart device through the gateway. This stage, illustrated in Figure 4, involves the following steps:

**At the Mobile User Side:** 

**Step 1:**  The mobile device generates a random number ($N_{v}$) and calculates the parameter ($N_{y}$).
  $N_{y}=N_{v}⊕K_{UG}$
**Step 2:**  The mobile device obtains its current location ($L_{C}$) and computes the parameter ($N_{C}$). With this parameter, the gateway can easily derive the current location using the shared secret key ($K_{UG}$) stored at the gateway. The mobile user also manages the session’s location history ($X_{n}$).
  $N_{C}=L_{C}⊕K_{UG}$
  $X_{n}=h\left(X_{n-1}∥L_{C}\right)$
**Step 3:**  The mobile user selects a smart device ($SID_{j}$) and computes the parameter ($SD_{q}$). The parameter $Y_{n}$ is the hash of the user’s location parameters and the entities’ identities.
  $Y_{n}=h(ID_{GW}∥TID_{U_{i}}∥L_{C}∥X_{n})$
  $SD_{q}=Y_{n}⊕SID_{j}⊕K_{UG}$
**Step 4:**  The mobile user computes the verification parameter ($V_{1}$) after generating the timestamp ($T_{1}$). Then, the mobile user sends the message ($M_{1}$) to the gateway.
  $V_{1}=h(TID_{U_{i}}∥K_{UG}∥ID_{GW}∥T_{1})$

**Message $M_{1}$ Passed from Remote User to Gateway** 
**At the Gateway Side:** 

**Step 1:**  Upon receiving the message ($M_{1}$), the gateway generates the timestamp ($T_{2}$). It checks the condition $T_{2}-T_{1}\leq \Delta T$ and verifies the $TID_{U_{i}}$ using its secret key (*k*) and the shared key ($K_{UG}$) derived from the parameter $N_{y}$. The gateway also checks the verification parameter ($V_{1}$).
  $N_{v}=K_{UG}⊕N_{y}$
  $V_{1}\overset{?}{=}h(TID_{U_{i}}∥K_{UG}∥ID_{GW}∥T_{1})$
**Step 2:**  After successfully verifying $V_{1}$, the gateway derives the current location from the parameter $N_{C}$ and recalculates the location history ($X_{n}$) using the previous location history value stored on the gateway from the previous session.
  $L_{C}=K_{UG}⊕N_{C}$
**Step 3:**  The gateway calculates the parameter $Y_{n}$ and compares the calculated value with the derived parameter $Y_{n}$ (from the user’s parameter $U_{G}$) to verify the mobile user based on their location parameters. Then, the targeted smart device identity is extracted from $SD_{q}$.
  $Y_{n}=K_{UG}⊕U_{G}∥TID_{U_{i}}$
  $Y_{n}\overset{?}{=}h(ID_{GW}∥TID_{U_{i}}∥L_{C}∥X_{n})$
  $SID_{j}=Y_{n}⊕SD_{q}⊕X_{n}$
**Step 4:**  After the above conditions are satisfied, the gateway computes the verification parameter $V_{2}$.
  $V_{2}=h(h\left(K_{GS}\right)∥SID_{j}∥ID_{GW}∥TID_{U_{i}}∥T_{2})$

**Message ($M_{2}$) Passed from Gateway to Smart Device** 
**At the Smart Device Side:** 

**Step 1:**  The smart device generates the timestamp ($T_{3}$) and compares it with the receiving time ($T_{2}$) of the message ($M_{2}$). It also verifies the verification parameter ($V_{2}$). All smart devices store their identities and the hash of their shared secret keys.
  $T_{3}-T_{2}\leq \Delta T$
  $V_{2}\overset{?}{=}h(h\left(K_{GS}\right)∥SID_{j}∥ID_{GW}∥TID_{U_{i}}∥T_{2})$
**Step 2:**  After successfully verifying $V_{2}$, the smart device computes the verification parameter $V_{3}$ and sends message $M_{3}$ to the gateway.
  $V_{3}=h(h\left(K_{GS}\right)∥SID_{j}∥ID_{GW}∥TID_{U_{i}}∥T_{3})$

**Message Passed from Smart Device to Gateway** 
**At the Gateway Side:** 

**Step 1:**  Upon receiving the message $M_{3}$, the gateway checks the condition $T_{4}-T_{3}\leq \Delta T$. It verifies the timestamp and the verification parameter $V_{3}$. If the verification fails, the session is terminated.
  $T_{4}-T_{3}\leq \Delta T$
  $V_{3}\overset{?}{=}h(h\left(K_{GS}\right)∥SID_{j}∥ID_{GW}∥TID_{U_{i}}∥T_{3})$
**Step 2:**  If the above conditions are satisfied, the gateway updates the temporary identity by encrypting the saved user identity ($ID_{U_{i}}$) with its secret key (*k*) along with a new random number ($r_{new}$).
  $TID_{U_{i(new)}}=E_{k}\left(ID_{U_{i}}∥r_{new}\right)$
**Step 3:**  The gateway computes the parameter $Z_{n}$ and the verification parameter $V_{4}$. It then sends the message ($M_{4}$) to the mobile user.
  $Z_{n}=K_{UG}⊕TID_{U_{i(new)}}$
  $V_{4}=h(N_{v}∥T_{4}∥K_{UG}∥Z_{n})$

**Message Passed from Gateway to Mobile User** 
**At the Mobile User Side:** 

**Step 1:**  The mobile user generates the timestamp ($T_{5}$) and compares it to the timestamp ($T_{4}$).
  $T_{5}-T_{4}\leq \Delta T$
**Step 2:**  The mobile user extracts the value of the new temporary identity ($TID_{U_{i(new)}}$) from the parameter $Z_{n}$ and verifies the verification parameter $V_{4}$.
  $TID_{U_{i(new)}}=Z_{n}⊕K_{UG}$
  $V_{4}\overset{?}{=}h(N_{v}∥T_{4}∥K_{UG}∥Z_{n})$
**Step 3:**  If the condition $TID_{U_{i(new)}}=TID_{U_{i}}$ is satisfied, the session is terminated. Otherwise, it implies that the mobile user has successfully authenticated the smart device. Finally, the mobile user updates the temporary identity.

## 5. Security Analysis of the Proposed Scheme

The security analysis of the proposed protocol is conducted to assess its strength and resilience against various attacks. The analysis is performed by considering a threat model (defined in Section 3) and employing BAN logic [18,19] and ProVerif [20,21].

### 5.1. Security Analysis with BAN Logic

BAN logic provides a set of defined rules for the formal analysis of authentication protocols [18]. It applies various logical rules to determine whether a protocol achieves its authentication goals [19]. The BAN logic notations are shown in Table 3. In the proposed scheme, eight goals are derived using BAN logic, as outlined below:

- Goal 1: $GWN|\equiv U_{i}\overset{TID_{U_{i}}}{⟷}$ GWN
- Goal 2: $GWN|\equiv U_{i}|\equiv U_{i}\overset{TID_{U_{i}}}{⟷}$ GWN
- Goal 3: $SID_{j}|\equiv GWN\overset{TID_{U_{i}}}{⟷}$$SID_{j}$
- Goal 4: $SID_{j}\left|\equiv GWN\right|\equiv GWN\overset{TID_{U_{i}}}{⟷}$$SID_{j}$
- Goal 5: $GWN|\equiv SID_{j}\overset{TID_{U_{i}}}{⟷}$ GWN
- Goal 6: $GWN|\equiv SID_{j}|\equiv SID_{j}\overset{TID_{U_{i}}}{⟷}$ GWN
- Goal 7: $U_{i}|\equiv GWN\overset{TID_{U_{i}}}{⟷}U_{i}$
- Goal 8: $U_{i}\left|\equiv GWN\right|\equiv GWN\overset{TID_{U_{i}}}{⟷}U_{i}$

The idealized form of the protocol is analyzed using BAN logic, and the results are as follows:

**Part 1: Idealized Protocol Form**

- M-1: $U_{i}\to$GWN: $TID_{U_{i}}$, $V_{1}$, $T_{1}$, $N_{y}:<N_{v}>KUG$, $SD_{q}:<Y_{n},SID_{j},X_{n}>$, $U_{G}:<Y_{n},TID_{U_{i}}>KUG$
- M-2: $GWN\to SID_{j}$: $TID_{U_{i}},V_{2},T_{2}$
- M-3: $SID_{j}\to$GWN: $V_{3},T_{3}$
- M-4: $GWN\to U_{i}$: $V_{4},T_{4},Z_{n}:<TID_{U_{i}\left(new\right)}>KUG$

**Part 2: Assumptions**

The following assumptions are considered for the analysis:

- A1: $U_{i}|\equiv \left(N_{v}\right)$
- A2: $GWN|\equiv (r_{i})$
- A3: $SID_{j}|\equiv \left(T_{3}\right)$
- A4: $GWN|\equiv SID_{j}\Rightarrow T_{3}$
- A5: $GWN|\equiv U_{i}\Rightarrow N_{v}$
- A6: $SID_{j}|\equiv GWN\Rightarrow T_{2}$
- A7: $SID_{j}|\equiv U_{i}\Rightarrow N_{V}$
- A8: $U_{i}|\equiv SID_{j}\Rightarrow T_{3}$
- A9: $U_{i}|\equiv GWN\Rightarrow r_{i}$

Using the BAN logic rules, the analysis proceeds as follows:

**Message 1:**

**M-1:** $U_{i}\to$GWN: $TID_{U_{i}}$, $N_{y}:<N_{v}>KUG$, $SD_{q}:<Y_{n},SID_{j},X_{n}>$, $T_{1}$ is $U_{i}$’s timestamp

By applying the Seeing rule, the following is obtained:

- S-1: $GWN⊲TID_{U_{i}},N_{y}:<N_{v}>KUG,T_{1},V_{1}$

By applying the Message Meaning rule and S-1, the following is obtained:

- S-2: $GWN|\equiv U_{i}|∼N_{v}$

By applying the Freshness Concatenation rule and S-2, the following is obtained:

- S-3: $GWN|\equiv U_{i}|\equiv N_{v}$

By applying the Jurisdiction rule and S-3, the following is obtained:

- S-4: $GWN|\equiv N_{v}$

By applying S-4 and the Session Key rule, the following is obtained:

- S-5: $GWN|\equiv U_{i}\overset{TID_{U_{i}}}{⟷}$ GWN **(Goal 1)**

By applying the Nonce Verification rule, the following is obtained:

- S-6: $GWN|\equiv U_{i}|\equiv U_{i}\overset{TID_{U_{i}}}{⟷}$ GWN **(Goal 2)**

**Message 2:**

**M-2:** $GWN\to SID_{j}$: $TID_{U_{i}},T_{2},V_{2}$. $T_{2}$ is the timestamp of GWN

By applying the Seeing rule, the following is obtained:

- S-7: $SID_{j}⊲TID_{U_{i}},T_{2},V_{2}$

By applying the Message Meaning rule and S-7, the following is obtained:

- S-8: $SID_{j}\left|\equiv GWN\right|∼T_{2}$

By applying the Freshness Concatenation rule and S-8, the following is obtained:

- S-9: $SID_{j}\left|\equiv GWN\right|\equiv r_{i}$

By applying the Jurisdiction rule and S-9, the following is obtained:

- S-10: $SID_{j}|\equiv r_{i}$

By applying S-10 and the Session Key rule, the following is obtained:

- S-11: $SID_{j}|\equiv GWN\overset{TID_{U_{i}}}{⟷}SID_{j}$ **(Goal 3)**

By applying the Nonce Verification rule and S-11, the following is obtained:

- S-12: $SID_{j}\left|\equiv GWN\right|\equiv GWN\overset{TID_{U_{i}}}{⟷}SID_{j}$ **(Goal 4)**

**Message 3:**

**M-3:** $SID_{j}\to$GWN: $V_{3},T_{3}$, $T_{3}$ is the timestamp of $SID_{j}$

By applying the Seeing rule, the following is obtained:

- S-13: $GWN⊲V_{3},V_{4},T_{3}$

By applying the Message Meaning rule and S-13, the following is obtained:

- S-14: $GWN|\equiv SID_{j}|∼T_{3}$

By using S-14 and the Freshness Concatenation rule, the following is obtained:

- S-15: $GWN|\equiv SID_{j}|\equiv T_{3}$

By applying the assumption S-15 and the Jurisdiction rule, the following is obtained:

- S-16: $GWN|\equiv T_{3}$

By applying S-16 and the Session Key rule, the following is obtained:

- S-17: $GWN|\equiv SID_{j}\overset{TID_{U_{i}}}{⟷}$ GWN **(Goal 5)**

By applying the Nonce Verification rule, the following is obtained:

- S-18: $GWN|\equiv SID_{j}|\equiv SID_{j}\overset{TID_{U_{i}}}{⟷}$ GWN **(Goal 6)**

**Message 4:**

**M-4:** $GWN\to U_{i}$: $V_{4},T_{4},Z_{n}:<TID_{U_{i}\left(new\right)}>KUG$, $T_{4}$ is the timestamp of GWN

By applying the Seeing rule, the following is obtained:

- S-19: $U_{i}⊲V_{4},Z_{n}<TID_{U_{i}\left(new\right)}>KUG,T_{4}$

By applying the Message Meaning rule and S-19, the following is obtained:

- S-20: $U_{i}\left|\equiv GWN\right|∼TID_{U_{i}\left(new\right)}^{\prime }$

By applying S-20 and the Freshness Concatenation rule, the following is obtained:

- S-21: $U_{i}\left|\equiv GWN\right|\equiv TID_{U_{i}\left(new\right)}$

By applying the Jurisdiction rule and S-21, the following is obtained:

- S-22: $U_{i}|\equiv TID_{U_{i}\left(new\right)}$

By applying the Session Key rule, the following is obtained:

- S-23: $U_{i}|\equiv GWN\overset{TID_{U_{i}\left(new\right)}}{⟷}U_{i}$ **(Goal 7)**

By applying the Nonce Verification rule, the following is obtained:

- S-24: $U_{i}\left|\equiv GWN\right|\equiv GWN\overset{TID_{U_{i}new}}{⟷}U_{i}$ **(Goal 8)**

After analyzing the scheme using BAN logic, it can be concluded that the proposed protocol achieves mutual authentication and securely establishes session key agreement.

### 5.2. Security Analysis with ProVerif

ProVerif is an automatic tool used for analyzing the security of cryptographic protocols [20]. It verifies that an attacker cannot extract sensitive data from encrypted messages as long as the key remains secret [21]. The detailed process of all queries and their respective results can be found in Table 4.

The following is the interpretation of the query-wise result of the ProVerif analysis.

- Query 1: The query “not attacker(TIDUinew[])” returns true, indicating that the new identity (TIDUinew) is secure from attacks.
- Query 2: The query “inj-event(end_U(IDUi[])) ==>inj-event(start_U(IDUi[]))” returns true, indicating that the connection functions securely for starting and closing on the user mobile.
- Query 3: The query “inj-event(end_GWN(IDGW[])) ==>inj-event(start_GWN(IDGW []))” returns true, indicating that the connection on the gateway node is securely opened and closed.
- Query 4: The query “inj-event(end_SD(SIDj[])) ==>inj-event(start_SD(SIDj[]))” returns true, indicating that the connection on the smart devices is securely opened and closed.

The ProVerif analysis confirms that the proposed protocol is secure and achieves the intended security properties of secrecy and authentication.

## 6. Informal Security Analysis

This section presents a security requirements analysis for user authentication protocols, focusing on the resistance to node capture attacks. Both general and specific functional and security requirements have been utilized to achieve the intended security properties of the schemes. Our proposed approach achieves all the security requirements, especially resistance to known attacks and node capture attacks, by comparing with the existing approaches [15,16,22,23,24], as shown in Table 5. Therefore, the rest of the discussion primarily focuses on how the proposed scheme withstands node capture attacks.

### 6.1. Resistance to Node Capture Attack

To evaluate the proposed user authentication protocol’s resilience against node capture attacks, we adopt the approach presented by Wang et al. [25]. The detailed explanation of each attack target is as follows:

#### 6.1.1. Mobile User (Attack Target)

Exploited Vulnerabilities → Attack Consequences

- Insecure Identity Transmission $\overset{Attack}{⟶}$ Break User Anonymity
  In the proposed protocol, the mobile user does not use its original identity but instead employs a temporary identity updated by the gateway in each session.
- Insecure Transmission of Secret Key $\overset{Attack}{⟶}$ Obtain Secret Key
  The mobile user does not directly transmit its shared secret key $K_{UG}$ in the exchanged messages. Instead, $K_{UG}$ is used to encrypt various parameters ($N_{Y}$, $N_{C}$, $U_{G}$, $V_{1}$) with the help of random numbers and other secret parameters. Therefore, the key $K_{UG}$ remains secure and cannot be extracted by an adversary.

#### 6.1.2. Smart Device (Attack Target)

- Improper Distribution of Secret Key $\overset{Attack}{⟶}$ Obtain Secret Key of All Target Smart Devices
  Each smart device possesses a unique shared secret key with the gateway. If a node capture attack compromises a smart device ($SID_{j}$), the adversary cannot compromise the shared secret key of other smart devices.
- Exposure of User’s Secret Parameter $\overset{Attack}{⟶}$ Impersonate the User
  During the authentication phase, the mobile user’s secret parameters are not forwarded in exchanged messages. These secret parameters encrypt the parameters exchanged over the public channel and a random number. If a compromised smart device attempts to compute the user’s secret parameters, it will fail to extract any relevant information. Hence, an adversary cannot impersonate the mobile user in the proposed protocol.
- Mobile User Fails to Identify Smart Devices $\overset{Attack}{⟶}$ Impersonation of All Smart Devices
  During the authentication phase, the mobile user selects the smart device to authenticate mutually. The mobile user possesses knowledge of the identities of all the smart devices connected to the network. Suppose the user fails to identify the smart device correctly based on its identity. In that case, it indicates that an adversary has either changed the identity of the smart device or the smart device is unresponsive when receiving authentication messages from the gateway. However, impersonating a compromised smart device does not lead to the impersonation of all smart devices within the system. This is due to each smart device’s unique shared secret keys.

#### 6.1.3. Gateway (Attack Target)

- Insecure Transmission of Secret Key $k\overset{Attack}{⟶}$ Break User Anonymity, Obtain Secret *k*
  The gateway, considered a secure entity in the proposed scheme, does not transmit its secret key *k* but uses it only for session key $K_{UG}$ computation. For the computation of exchanged messages, the gateway employs the shared secret keys ($K_{UG}$, $h(K_{GS})$).

#### 6.1.4. Session Key (Attack Target)

- Forward Secrecy Issue $\overset{Attack}{⟶}$ Obtain Previous Session Key of $SID_{j}$
  The proposed scheme achieves forward secrecy, as discussed in the security requirements above. An adversary cannot derive the session key computation from a previous session since only the trusted entity, the gateway, can compute the session key.
- Improper Distribution of Smart Device Secret Keys $\overset{Attack}{⟶}$ Obtain Previous Session Key of All Smart Devices
  With its unique identity, each smart device must be registered with the gateway before joining the environment. The gateway distributes a unique secret key corresponding to each smart device’s identity. Additionally, the session key is updated during each session. Consequently, even if an adversary manages to capture a node and obtain the session key, it does not compromise the security of the entire system.

#### 6.1.5. Availability (Attack Target)

- Insecure Transmission of Updated Session Key $\overset{Attack}{⟶}$ Modify Session Key
  The gateway entity updates the session key using its secret key. The new session key ($TID_{U_{i}\left(new\right)}$) is transmitted to the user by encrypting it with the shared secret key ($K_{UG}$). Only the user can obtain the session key by decrypting it with $K_{UG}$. As a result, an adversary cannot access or modify the updated session key, ensuring its integrity.

## 7. Performance Analysis of the Proposed Protocol

This section compares the proposed protocol with previously proposed security protocols [15,16,22,23,24] in terms of communication and computation costs [26].

### 7.1. Communication Costs Analysis

The comparison of communication cost is shown in Table 6. Communication cost refers to the number of bits and messages exchanged during a single scheme transaction. The bits and messages are calculated based on the approximate values of functions and parameters used in the proposed protocol [27]. The following are the values of the functions and parameters: ECC point value: 320 bits, hash digest (SHA-1) value: 160 bits, nonce/identities value: 128 bits, timestamp value: 32 bits, random number value: 64 bits. In the proposed protocol, four messages are exchanged: message $M_{1}$ transmitted with 160 bytes, message $M_{2}$ transmitted with 40 bytes, message $M_{3}$ transmitted with 36 bytes, and message $M_{4}$ transmitted with 47.

The proposed protocol exhibits lower communication costs than the mentioned protocols, except for the Fakroon et al. [24] scheme. Although Fakroon et al. have lower communication costs than the proposed protocol, they fail to provide the required general security requirements. In contrast, the proposed protocol satisfies the necessary security requirements for IoT smart home systems.

### 7.2. Computation Costs Analysis

The comparison of computation cost is shown in Table 7. The computation costs of the protocols are calculated for each party involved, including the smart user, gateway, and smart device. The computation cost of the proposed protocol is calculated as follows: $4h_{U_{i}}+4h_{GWN}+2SE_{GWN}+1h_{SD}=9h+2S_{ED}$. Table 8 shows the computation cost of proposed approaches compared to the state-of-the-art approaches [15,16,22,23,24].

The computation time experiment by Kilinc and Yanik [28] is used to calculate computational time. The experiment was conducted on the Ubuntu operating system with an Intel dual-core Pentium processor, with specifications including a 2.20GHz processor and 2048MB RAM. According to the experiment, the computational time of different cryptographic primitives is as follows: time for hash ($T_{h}$) is 0.0023 ms, time for bilinear function ($T_{B}$) is 5.811 ms, time for MAC ($T_{MAC}$) is 0.0046 ms, time for modular exponentiation ($T_{me}$) is 3.8500 ms, and time for encryption/decryption ($T_{k}$) is 0.0046 ms.

The execution/running time of the proposed protocol is 0.0299 ms. The comparison of the computational cost of the proposed approach with respect to the state-of-the-art approaches [15,16,22,23,24] is given in Table 8. According to the experimental results, the proposed approaches outperform all the previous approaches.

## 8. Conclusions and Future Work

This paper comprehensively analyzed state-of-the-art user-authentication schemes in the context of smart home systems. Our analysis identified several limitations and security vulnerabilities in existing schemes, highlighting the need for an improved solution. To address these shortcomings, we propose a secure and enhanced user-authentication scheme tailored for smart home environments. We performed a thorough security analysis of our protocol using formal computational models such as BAN logic and ProVerif tools. The evaluation demonstrated that our scheme effectively mitigates various security vulnerabilities, providing robust protection against attacks. Furthermore, we conducted a performance analysis to assess the computational and communication costs of the proposed scheme. The results indicated that our protocol achieves efficiency in resource utilization, making it suitable for deployment in IoT-based smart home environments.

Our future work will primarily focus on the dynamic aspects of user authentication within smart home environments. This entails exploring adaptive authentication mechanisms capable of accommodating changes in user profiles, roles, and permissions within the smart home system. Additionally, we plan to investigate techniques to improve the scalability and interoperability of user-authentication schemes, facilitating seamless integration with a diverse array of smart home devices and platforms. By addressing these areas, our objective is to bolster the security, usability, and flexibility of user authentication in smart homes. Ultimately, we aim to contribute to developing robust and efficient authentication solutions for future IoT applications, thereby safeguarding the privacy and security of smart home users.

## Figures and Tables

**Table 1:** Comparative analysis of user-authentication schemes for IoT-based smart home systems.

Scheme	Mutual Authentication	User Anonymity	Untraceability	Vulnerabilities
Vaidya et al. [11]	Yes	No	No	Password-guessing, user-impersonation
Kim et al. [12]	Yes	No	No	Password-guessing, user impersonation
Li [13]	No	No	No	Computation overhead, lack of mutual authentication
Santoso et al. [14]	Yes	No	No	Insider attacks, lack of user anonymity
Kumar et al. [15]	No	No	No	Lack of mutual authentication, user anonymity
Wazid et al. [16]	Yes	No	No	Synchronization attacks

**Table 2:** Notation guide of proposed protocol.

Notations	Description
$ID_{U_{i}}$	User Identity
$U_{i}$	User
$TID_{U_{i}}$	Temporary Identity
$K_{UG}$	Shared Keys between Gateway and Mobile User
$K_{GS}$	Shared Keys between Gateway and Smart Device
k	Secret key of Gateway
t	Timestamp
$L_{C}$	Current Location
$X_{n}$	History of Location
$V_{1}$, $V_{2}$, $V_{3}$, $V_{4}$	Verification Parameter
$SD_{i}$	Smart Device
$SID_{j}$	Smart Device Identity
$ID_{GW}$	Gateway Identity
$TID_{U_{i(new)}}$	New Temporary Identity
$r_{U_{i}}$,$N_{H}$,$r_{U_{i}}$,$N_{v}$	Random Numbers
⊕	The exclusive XOR Operation
||	Concatenation
h	Hash

**Table 3:** Notation Guide for Ban Logic.

Notations	Description
P|≡X	P believes on X
P⊲X	P sees that X
P|∼X	P once said X
P⇒X	P has total jurisdiction on X
#(X)	X is updated and fresh
(X,Y)	x,y is component of formula(x,y)
${\left(X\right)}_{k}$	Hash of message X using a key K
$<X{>}_{y}$	X is combined with y
$P\overset{k}{⟷}Q$	P and Q are using shared key K for
	communication process
$TID_{U}i$	Session key $TID_{U}i$ is used one time in a
	current session
$\frac{P|\equiv P\overset{K}{⟷}Q.p⊲<X{>}_{K}}{P|\equiv Q|∼X}$	Message Meaning rule
$\frac{P|\equiv \#(X)}{P|\equiv \#(X,Y)}$	Freshness Concatenation rule
$\frac{P|\equiv \#(X),P|\equiv Q|∼X}{P|\equiv Q|\equiv X}$	Nonce verification
$\frac{P|\equiv Q\Rightarrow X,P|\equiv Q|\equiv X}{P|\equiv X}$	Jurisdiction rule

**Table 4:** Security analysis through ProVerif.

Query	ProVerif Response
1–Query inj-event(end_U(TIDUinew[])) ==\textgreater inj-event(start_U(TIDUinew[]))	
Completing…Starting query not attacker(TIDUinew[])	RESULT not attacker(TIDUinew[]) is true.
2–Query inj-event(end_U(IDUi[])) ==>inj-event(start_U(IDUi[]))	
Completing…	
Starting query inj-event(end_U(IDUi[])) ==>inj-event(start_U(IDUi[]))	&RESULT inj-event(end_U(IDUi[]))
&==>inj-event(start_U(IDUi[])) is true.	
3–Query inj-event(end_GWN(IDGW[]))==>inj- event(start_GWN(IDGW[]))	
Completing…	
Starting query inj-event(end_GWN(IDGW[])) ==>inj-event(start_GWN(IDGW[]))	&RESULT inj-event(end_GWN(IDGW[]))
&==>inj-event(start_GWN(IDGW[])) is true.	
4–Query inj-event(end_SD(SIDj[]))==\>inj- event(start_SD(SIDj[]))	
Completing…	
Starting query inj-event(end_SD(SIDj[])) ==>inj-event(start_SD(SIDj[]))	&RESULT inj-event(end_SD(SIDj[]))
&==>inj-event(start_SD(SIDj[])) is true.	

**Table 5:** Security comparison table.

Requirements	[15]	[16]	[22]	[23]	[24]	Proposed Scheme
F1	×	√	√	√	√	√
F2	√	√	√	√	√	√
F3	√	√	√	×	√	√
F4	×	×	×	×	√	√
S1	×	√	×	√	×	√
S2	×	×	√	×	×	√
S3	×	×	×	√	×	√
S4	√	×	×	×	×	√
S5	×	×	×	×	×	√

F1: Mutual Authentication, F2: Key Agreement, F3: Sound Repairability, F4: No Location Tracking, S1: User Anonymity, S2: Forward Secrecy, S3: No Password Exposure, S4: Resistance to known Attack, S5: Resistance to Node Capture Attack; √: Yes provides, ×: Does not provide.

**Table 6:** Comparison of communication costs of the protocols.

Protocols	$M_{1}$	$M_{2}$	$M_{3}$	$M_{4}$	Total-Bytes	Messages
Kumar et al. [15]	512	448	192	-	1152	3
Wazid et al. [16]	60	120	64	160	404	4
Shuai et al. [22]	108	84	36	68	296	4
Banerjee et al. [23]	52	84	52	100	288	4
Fakroon et al. [24]	92	56	56	56	260	4
Proposed Protocol	160	40	36	47	283	4

**Table 7:** Comparison of computation costs of the protocols.

Computation Cost	${\mathrm{Total}}_{\mathrm{CC}}$
[15]	4$T_{S_{ED}}$+ $1T_{hmac}$+ 4$T_{h}$
[16]	22$T_{h}$+ 4$T_{S_{ED}}$ + $T_{fe}$
[22]	16$T_{h}$ + 3$T_{m}$
[23]	24$T_{h}$+ 1$T_{b}$
[24]	33$T_{h}$
Proposed Scheme	9$T_{h}$ + 2$T_{S_{ED}}$

$U_{i_{CC}}$: Computation Cost of Mobile User, $GWN_{CC}$: Computation Cost of Gateway, $SD_{CC}$: Computation Cost of Smart Device, $Total_{CC}$: Total Computation Costs.

**Table 8:** Comparison of computation costs in milliseconds.

Computation Cost	Total Cost in Milliseconds
[15]	0.0322 ms
[16]	0.0736 ms
[22]	11.586 ms
[23]	5.866 ms
[24]	0.0759 ms
Proposed Scheme	0.029 ms

## Data Availability

Available upon request.
